# Risk factors of neurological deficit and pulmonary cement embolism after percutaneous vertebroplasty

**DOI:** 10.1186/s13018-019-1459-4

**Published:** 2019-11-29

**Authors:** Ming-Kai Hsieh, Fu-Cheng Kao, Ping-Yeh Chiu, Lih-Huei Chen, Chia-Wei Yu, Chi-Chien Niu, Po-Liang Lai, Tsung-Ting Tsai

**Affiliations:** 1 Department of Orthopedic Surgery, Chang Gung Memorial Hospital, Taoyuan, Taiwan; 2 Bone and Joint Research Center, Chang Gung Memorial Hospital, Linkou, Taiwan; 3 College of Medicine, Chang Gung University, Taoyuan, Taiwan

**Keywords:** Bone cements, Iatrogenic disease, Adverse effects, Paresis, Pulmonary embolism

## Abstract

**Background:**

The risk factors, incidence, and clinical management of pulmonary cement embolism and neurological deficit during percutaneous vertebroplasty (PVP) were evaluated.

**Methods:**

Three thousand one hundred and seventy-five patients with symptomatic osteoporotic vertebral compression fractures (OVCFs) treated with PVP were retrospectively reviewed in a single institution. Clinical parameters such as age, gender, number of fractures, and time from fracture to vertebroplasty were recorded at the time of surgery. Image and surgical parameters including the amount of cement, the vertebral level, uni- or bipedicle surgical approach, and leakage pattern were recorded.

**Results:**

Type-C leakage, including paraspinal (25%), intradiscal (26%), and posterior (0.7%) leakage, was more common than type-B (11.4%) and type-S leaks (4.9%). Cement leakage into the spinal canal (type-C posterior) occurred in 26 patients (0.7%), and four patients needed surgical decompression. Three in nine patients with leakage into thoracic spine needed decompressive surgery, but only one of 17 patients into lumbar spine needed surgery (*p* < 0.01). Age, gender, number of fractures, and time from fracture to vertebroplasty were not risk factors of pulmonary cement embolism or neurological deficit. The risk factor of pulmonary cement embolism was higher volume of PMMA injected (*p* < 0.001) and risk factor of neurological deficit was type-C posterior cement leakage into thoracic spine. The incidence of pulmonary cement embolism was significantly high in the volume of PMMA injected (PMMA injection < 3.5 cc: 0%; 3.5–7.0 cc: 0.11%; > 7.0 cc: 0.9%; *p* < 0.01) which needed postoperative oxygen support.

**Conclusions:**

Cement leakage is relatively common but mostly of no clinical significance. Percutaneous vertebroplasty in thoracic spine and high amount of PMMA injected should be treated with caution in clinical practice.

## Background

With the aging of the population, the incidence of osteoporotic vertebral compression fractures (OVCFs) is increasing and is becoming a major healthcare concern [1].

Percutaneous vertebroplasty (PVP) has gained widespread acceptance and is implemented broadly, mainly as a treatment method for painful OVCFs [2–6]. Its benefit over conservative treatment has been reported in a large and high-quality randomized clinical trial [7]. However, there are also reports that put into doubt the efficacy of vertebroplasty as a treatment for VCFs. Some studies have found that PVP had no efficacy compared with sham-operated patients [8–10]. Later reports by the same authors showed that vertebroplasty had no or limited value in long-term follow-up studies [10–12].

The complication rate of PVP is low, and has been reported to be 1.6% to 3.8% in meta-analyses [13, 14]. Severe complications of PVP are rare; they are restricted to case reports and mainly comprise sequelae of excessive cement leakage, such as paraplegia [15], neurologic deficits [16, 17], cardiac perforation [18, 19], and even death [20].

The rate of occurrence of cement leakage itself appears to vary, with reported incidences ranging from less than 5% to more than 80% [21–24]. When assessed using computed tomography (CT) scanning, which is known to be substantially superior to intraoperative fluoroscopy or postoperative radiography for detection of cement leakage [24, 25], the incidence of leakage was found to be 63% to 87% [21–25].

PVP can also increase the risk of fractures of adjacent vertebrae [26–30]. The incidence of postoperative infections in patients undergoing PVP is low [31].

In this study, we analyzed clinically significant complications after cement leakage in 3175 patients treated with PVP for symptomatic OVCFs.

## Methods

### Patients

We retrospectively reviewed the records of 3175 patients treated with PVP for symptomatic OVCFs at our institution between 2001 and 2011. The average number of patients treated per year was 288 (63–381 patients per year). The Chang Gung Medical Foundation Institutional Review Board approved this study (IRB No. 103-3388B) and waived the requirement for informed consent due to the retrospective nature of the study. All patients met the following criteria: (1) focal midline back pain managed inadequately with appropriate conservative treatment, (2) back pain related to VCF location on radiography, and (3) the presence of bone marrow edema on magnetic resonance imaging (MRI), as indicated by a hypointense signal on T1-weighted images and a hyperintense signal on T2-weighted images. Exclusion criteria were active infection, neurologic deficit, pathological fractures, and uncorrected therapeutic anticoagulation.

Clinical parameters such as age, gender, and time from fracture to vertebroplasty were recorded at the time of surgery. Parameters related to imaging and technical characteristics, including the amount of bone cement injected per procedure, the vertebral level of the fracture, surgical approach (uni- or bipedicle), filling pattern, and any leakage of cement, which was classified into three types [25], as follows: B, via the basivertebral vein; S, via the segmental vein; and C, through a cortical defect, were also recorded.

### Surgical technique

Percutaneous vertebroplasty was performed under local or general anesthesia with the patient in a prone position on a radiolucent table with his/her spine extended by chest and pelvic bolsters. A preoperative prophylactic single-dose antibiotic was administered to each patient, and fluoroscopy was used throughout the procedure. A 1-cm incisional wound was made on the pedicle level of the skin; the correct incision site was identified using the anteroposterior view fluoroscopically. An 11- or 13-gauge vertebroplasty needle was gently hammered into the anterior third of the vertebral body, followed by injection of polymethylmethacrylate (PMMA) bone cement (SimplexP; Stryker Howmedica Osteonics, Allendale, NJ, USA), until a satisfactory distribution of the cement, that is, a symmetrical filling of the central and anterior parts of the vertebral body, was obtained, or until cement leakage was noted. When necessary, a second needle was advanced into the vertebral body through the contralateral pedicle, followed by injection of the cement.

### Statistical analysis

The imaging and operative data were analyzed using the SPSS statistical software package (SPSS statistical software 16.0; SPSS Inc., Chicago, IL, USA). Comparisons between groups were performed with the use of the t test, and *P* values < 0.05 were deemed significant.

## Results

This study included 2127 (67%) women and 1048 men (33%), 60 to 98 years old (mean age, 76 years), all with at least 6 months of follow-up. A total of 3812 vertebroplasty procedures were performed in 3175 patients, among which 2644 had a single fracture, 436 had two vertebral fractures, 84 had three fractures, and 11 had four fractures (Table 1). Most operations were performed in subacute stage (56.7%), and chronic stage (40.7%); only 2.6% in acute stage (Table 1). Patient number of cement leakage corresponded to each characteristic were detailed listed (Table 1). There was no significantly statistical difference of types of cement leakage between age, gender, number of fracture treated, and time from fracture to vertebroplasty.

**Table 1:** Patient demographics and clinical data

Characteristic	Number (%)	Cement B type (*n* = 380)	leakage S type (*n* = 160)	C type-paraspinal (*n* = 790)	C type-intradiscal (*n* = 835)	C type-posterior (*n* = 26)	*p* value
Age (year) (*n* = 3175)							
60–69	594 (18.7%)	69	33	148	156	5	*p* > 0.05
70–79	1254 (39.5%)	148	60	312	330	10	
80–89	1118 (35.2%)	138	56	278	293	9	
90–99	209 (6.4%)	25	11	51	56	2	
Gender (*n* = 3175)							
Female	2127 (67%)	254	106	529	557	17	*p* > 0.1
Male	1048 (33%)	126	54	261	278	9	
No. of fractures treated (*n* = 3175 patients)							
1	2644 (83.2%)	133	133	658	693	19	*p* > 0.05
2	436 (13.7%)	55	22	109	117	4	
3	84 (2.6%)	11	4	21	22	2	
4	11 (0.5%)	1	0	3	3	1	
Time from fracture to vertebroplasty (*n* = 3175 patients)							
Acute (0–2 weeks)	82 (2.6%)	9	3	20	21	1	*p* > 0.05
Subacute (2 weeks–3 months)	1801 (56.7%)	215	88	448	472	15	
Chronic (> 3 months)	1292 (40.7%)	156	69	321	342	10	

Parameters related to imaging and technical characteristics, including the method of approach, amount of bone cement injected, and local or general anesthesia used were recorded (Table 2). Most operation were performed using unipedicular approach (98.4%) and bipedicular procedures were performed if poor filling of vertebra after unipedicular approach. The volume of cement injected were less than 3.5 cc in 320 patients (8.4%), 3.5 cc to 7 cc in 2821 patients (74%), and more than 7 cc in patients (17.6%). General anesthesia were used only in 153 patients (4.8%) with poor surgical compliance. The levels treated were from T5 to L5; four in T5, ten in T6, eight in T7, 19 in T8, 21 in T9, 36 in T10, 72 in T11, 1170 in T12, 1187 in L1, 601 in L2, 304 in L3, 272 in L4, and 108 in L5; with an emphasis on T12 and L1 (62%).

**Table 2:** Parameters related to imaging and technical characteristics

Operative characteristic (*n* = 3812 vertebra)	Number (%)
Unipedicular approach	3751 (98.4%)
Bipedicular approach	61 (1.6%)
Volume injected	
< 3.5 cc	320 (8.4%)
3.5–7 cc	2821 (74%)
> 7 cc	671 (17.6%)
Anesthesia	
Local	3022 (95.2%)
General	153 (4.8%)

### Cement leakage condition

Of the total 3812 vertebroplasty procedures performed, cement leakage was found in 2542 vertebrae (66.7%) (Table 3). A detailed demonstration of characters and appearance of cement leakage were also demonstrated in Table 3. Type-B leaks (11.1%) via the epidural venous plexus and type-S leaks (3.9%) via the segmental vein were less common than type-C leaks (51.8%). Type-C leaks, which occurred via a cortical defect, were divided into three groups: type-C paraspinal (24.9%), indicating leaks around a vertebral body; type-C intradiscal (26.2%), indicating leakage into an adjacent disc; and type-C posterior (0.7%), indicating leaks into the spinal canal (Table 3).

**Table 3:**
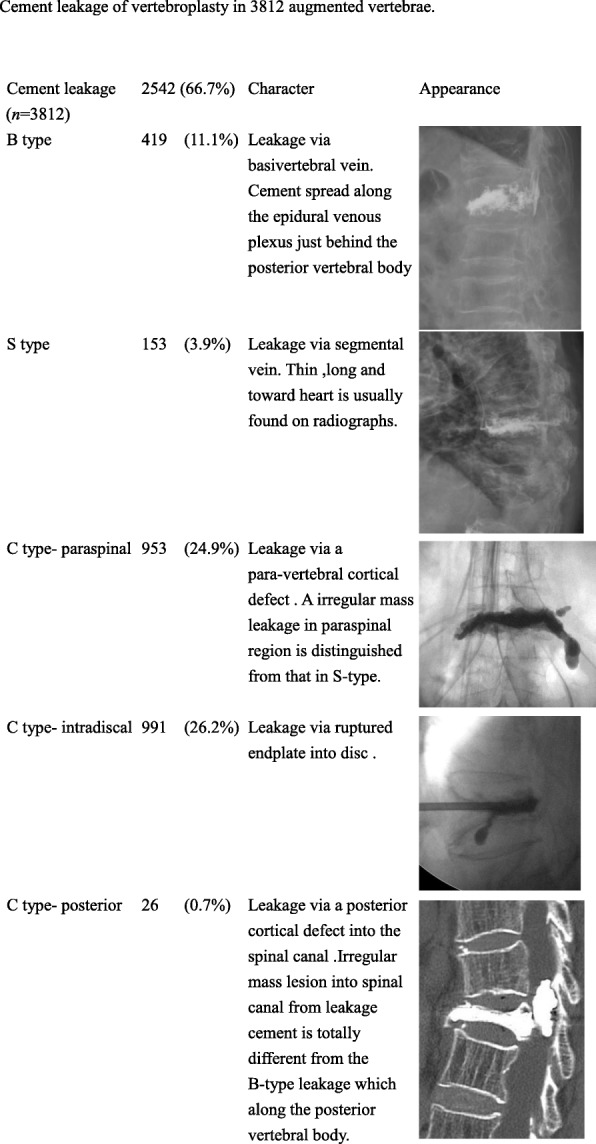
Cement leakage of vertebroplasty in 3812 augmented vertebrae

**Fig. 1:**
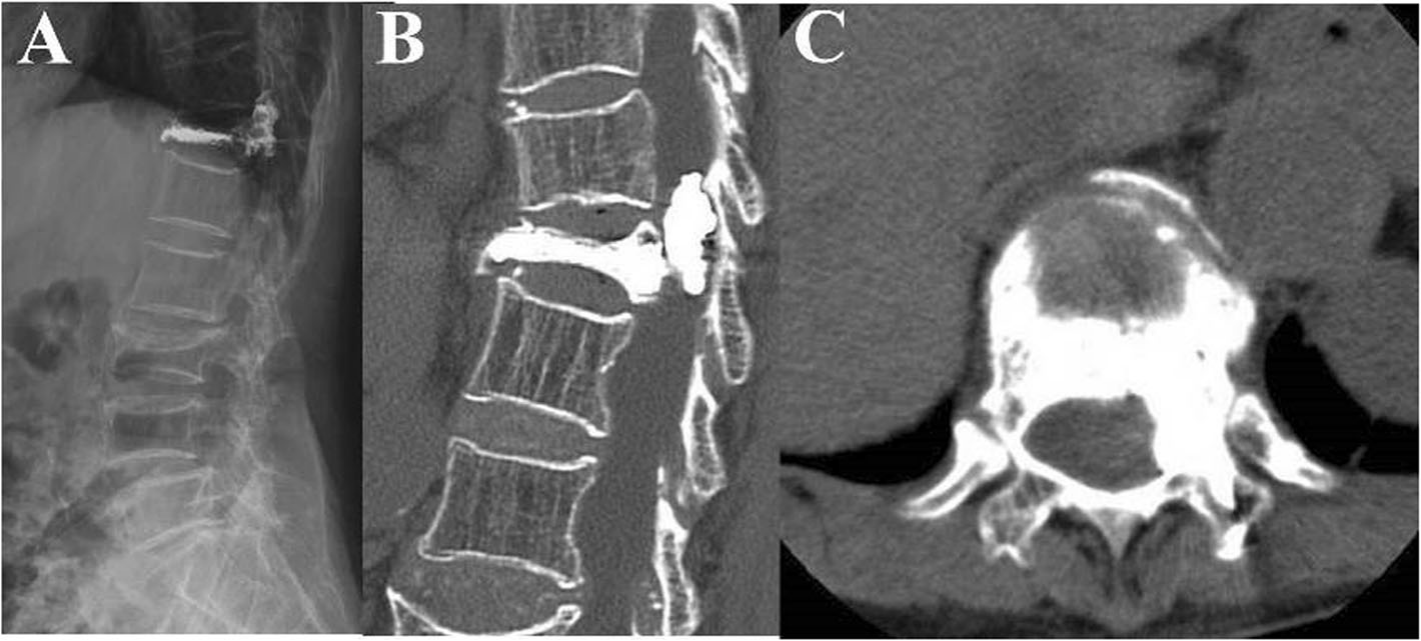
An 89-year-old female developed severe back pain and left-side lower limb motor weakness after T12 PVP using PMMA cement. Postoperative lateral (**a**) and CT (**b**) showing a type-C posterior leakage and cement leakage into the left spinal canal, which caused left-side lower limb weakness (**c**). The neurological deficit was fully recovered after immediate decompression surgery

**Fig. 2:**
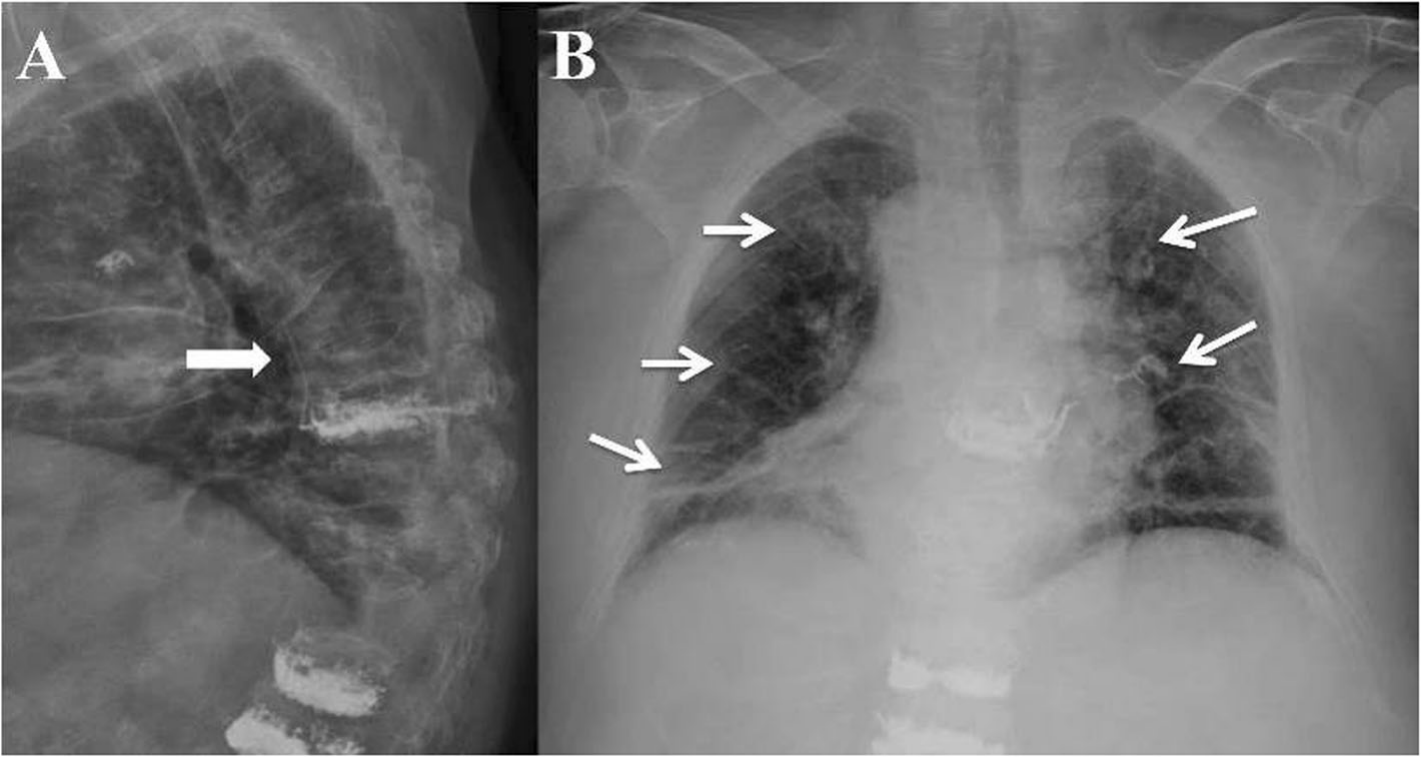
Postoperative lateral spinal radiograph 1 day after PVP with PMMA of osteoporotic fractures at levels T8, T12, and L1 in an 86-year-old male. The patient had dyspnea and chest pain after operation; type S leakage along the paravertebral veins (arrow) was noted (**a**). High-density PMMA cement with a tubular shape and branching opacities (arrows) distributed throughout the lungs and corresponding to lung vessels, caused pulmonary embolism, as seen on chest radiograph (**b**). After oxygen support and anticoagulation therapy for 3 days, follow-up revealed no symptom aggravation

Clinically significant complications were noted in 13 augmented vertebrae—four of 26 patients with a type-C posterior leak needed decompression surgery (Fig. 1) due to canal compromise and nine of 153 patients with a type S leak needed postoperative oxygen support due to pulmonary embolism (Fig. 2).

Incidence of cement leakage and pulmonary embolism was the same in both the thoracic and lumbar areas (Table 4), but there was a statistical difference in neurological deficits between these two locations. Three of nine patients with a T-spine type-C posterior leak needed decompression surgery due to canal compromise, but only one of 17 patients with an L-spine type-C posterior leak needed another surgery (*p* < 0.01) (Table 4).

**Table 4:** Cement leakage and complications during percutaneous vertebroplasty according to thoracic and lumbar spine

Location	T-spine (*n* = 1340)	L-spine (*n* = 2472)	*P* value
Cement leakage			
B type	130 (9.7%)	289 (11.7%)	*p* > 0.05
S type	61 (4.5%)	92 (3.7%)	*p* > 0.05
C type- paraspinal	381 (28.4%)	572 (23.1%)	*p* > 0.05
C type- intradiscal	289 (21.6%)	702 (28.3%)	*p* > 0.05
C type- posterior	9 (0.67%)	17 (0.69%)	*p* > 0.05
Clinical complication			
Neurological deficit	3 (0.22%)	1 (0.04%)	*p* < 0.01
Pulmonary embolism	3 (0.22%)	6 (0.24%)	*p* > 0.05

The volume of PMMA injected was highly correlated with type S leakage and pulmonary cement embolism (PMMA injection < 3.5 cc: 0%; 3.5–7.0 cc: 0.11%; > 7.0 cc: 0.9%; *p* < 0.01) which needed postoperative oxygen support. There was no difference in other types of leakage or neurological deficits (Table 5).

**Table 5:** Cement leakage and complications according to volume of cement injected

Volume of cement injected	< 3.5 cc (*n* = 320)	3.5–7.0 cc (*n* = 2821)	> 7.0 cc (*n* = 671)	*P* value
Cement leakage				
B type	22 (6.8%)	310 (11%)	87 (12.9%)	*p* > 0.05
S type	9 (2.8%)	81 (2.9%)	63 (9.4%)	*p* < 0.01
C type- paraspinal	64 (20%)	790 (28%)	99 (14.8%)	*p* > 0.05
C type- intradiscal	75 (23.4%)	730 (25.8%)	186 (27.7%)	*p* > 0.05
C type- posterior	2 (0.62%)	19 (0.67%)	5 (0.74%)	*p* > 0.05
Clinical complication				
Neurological deficit	0 (0%)	3 (0.11%)	1 (0.15%)	*p* > 0.05
Pulmonary embolism	0 (0%)	3 (0.11%)	6 (0.9%)	*p* < 0.01

## Discussion

Osteoporosis is a systemic disease that results from progressive bone mineral loss and changes in bony component, leaving the spinal vertebrae vulnerable to VCFs. Painful osteoporotic VCFs can be a devastating burden for patients, as they impair physical function and quality of life. Moreover, VCFs can lead to progressive sagittal spine deformities and changes in spinal biomechanics, which are believed to contribute to a five-fold increased risk of further fracture [32]. Conservative treatment for the pain caused by VCFs includes analgesic medication, bed rest, and back braces; however, these therapies do not address spinal deformities [1]. Furthermore, pain and disability may be prolonged while the fractured vertebral body heals [33]. PVP in osteoporotic VCFs, involving the percutaneous injection of cement directly into the fractured vertebra, is effective in ameliorating VCF-associated pain [34–36]. The limitation of this procedure is the substantial risk of extravertebral cement leakage after high-pressure cement injection, with severe clinical complications including spinal cord compression [15, 37–40], radicular pain [41], and systemic embolism [42–46]. Local leakage of PMMA is frequent, and varies up to more than 70% [1, 47–51]; however, in most cases, the leakage does not produce any symptoms [47–49].

In our review, cement leaks were noted in 69.1% of all performed procedures, but only 0.72% of patients had clinical complications. These included neurological deficit in four patients (0.26%) who achieved partial or full recovery after decompression surgery, and pulmonary embolism in nine patients (0.46%) who needed postoperative oxygen support.

There were statistical differences in neurological deficits between the thoracic and lumbar spine (Table 4), leading us to believe that cement leakage is not tolerated in the narrowing thoracic spinal canal. It was of interest that only four of 26 patients with a type-C posterior leak had clinical symptoms. We thought that the kyphotic spinal curvature might have made the canal wider. The four patients with a neurological deficit achieved partial or full recovery after decompression surgery.

In our series, the nine patients with symptomatic pulmonary embolism were all in the type-S leak group (Table 5), and this may be related to the cement embolism pathophysiology. The PMMA flows out through the basivertebral vein and then through the anterior external vertebral venous plexus, which leads to the pulmonary veins via the segmental spinal veins, the vena radicularis magna, the azygos vein, and the accessory hemiazygos vein [52]. A symptomatic pulmonary embolism following vertebroplasty can occur either by migration of acrylic or migration of fat and bone marrow cells [53]. On the one hand, the majority of radiologically detected PMMA migration into lung vessels is asymptomatic and, on the other, fat embolism is more frequent than PMMA embolism [54]. According to the observation, in cases of symptomatic pulmonary embolism with detectable PMMA in lung vessels after vertebroplasty, fat embolism occurring simultaneously may also contribute to the clinical manifestation of pulmonary embolism. Early symptoms caused by the passing of emboli through the pulmonary artery occur 6 s after injection, which should be distinguished from the very rapid decrease in heart rate and the fall in arterial pressure caused by a nerve reflex response occurs about 2 s after injection.

One study [55] recommended no treatment besides clinical follow-up for asymptomatic patients with peripheral pulmonary cement embolism, and initial heparinization and a following 6-month coumarin therapy for symptomatic pulmonary cement embolism.

Pulmonary embolism occurs in 4–26% of patients [56–59], but it occurred in only nine patients in our study. While symptoms were resolved after oxygen support and anticoagulation therapy, as recommended [60], no patient required open cardiac surgery to remove the cement from the lungs or from the right perforated ventricle.

Two limitations were in our study. First, this is a retrospective single center study. Second, only X-ray image was not enough to detect cement leakage and result in under estimate the leakage rate.

## Conclusion

PVP with PMMA is a common and effective treatment modality for painful osteoporotic vertebral compression fractures, but is not as simple and risk-free as suggested in many radiology and spinal surgery studies. Vertebroplasty is a minimally invasive surgical procedure with a high rate of cement leakage (69.1%), but a low incidence of clinical complications (0.72%). When injecting a high volume of cement, physicians should be aware of type S leaks if there are signs of pulmonary embolism, including decreased blood pressure, tachycardia, and dyspnea. Awareness of possible neurological symptoms should be heightened when there is cement leakage into the thoracic spinal canal.

## Data Availability

The data that support the findings of this study are available from Chang Gung Memorial Hospital but restrictions apply to the availability of these data, which were used under license for the current study, and so are not publicly available. Data are however available from the authors upon reasonable request and with permission of Chang Gung Memorial Hospital.

## Abbreviations

OVCF: Osteoporotic vertebral compression fracture
PVP: Percutaneous vertebroplasty
